# Exercise tolerance and quality of life in hemodynamically partially improved patients with chronic thromboembolic pulmonary hypertension treated with balloon pulmonary angioplasty

**DOI:** 10.1371/journal.pone.0255180

**Published:** 2021-07-23

**Authors:** Kotaro Miura, Yoshinori Katsumata, Takashi Kawakami, Hidehiko Ikura, Toshinobu Ryuzaki, Yasuyuki Shiraishi, Shogo Fukui, Michiyuki Kawakami, Takashi Kohno, Kazuki Sato, Keiichi Fukuda

**Affiliations:** 1 Department of Cardiology, Keio University School of Medicine, Tokyo, Japan; 2 Institute for Integrated Sports Medicine, Keio University School of Medicine, Tokyo, Japan; 3 Department of Rehabilitation Medicine, Keio University Hospital, Tokyo, Japan; 4 Department of Cardiovascular Medicine, Kyorin University School of Medicine, Mitaka, Tokyo, Japan; Vanderbilt University Medical Center, UNITED STATES

## Abstract

The efficacy of extensive balloon pulmonary angioplasty (BPA) beyond hemodynamic improvement in chronic thromboembolic pulmonary hypertension (CTEPH) patients has been verified. However, the relationship between extensive BPA in CTEPH patients after partial hemodynamic improvement and exercise tolerance or quality of life (QOL) remains unclear. We prospectively enrolled 22 CTEPH patients (66±10 years, females: 59%) when their mean pulmonary artery pressure initially decreased to <30 mmHg during BPA sessions. Hemodynamic and echocardiographic data, cardiopulmonary exercise testing, and QOL scores using the 36-item short form questionnaire (SF-36) were evaluated at enrollment (entry), just after the final BPA session (finish), and at the 6-month follow-up (follow-up). We analyzed whether extensive BPA improves exercise capacity and QOL scores over time. Moreover, the clinical characteristics leading to improvement were elucidated. The peak oxygen uptake (VO_2_) showed significant improvement at entry, finish, and follow-up (17.3±5.5, 18.4±5.9, and 18.9±5.3 mL/kg/min, respectively; *P*<0.001). Regarding the QOL, the physical component summary (PCS) scores significantly improved (32±11, 38±13, and 43±13, respectively; *P*<0.001), but the mental component summary scores remained unchanged. Linear regression analysis revealed that age and a low peak VO2 at entry were predictors of improvement in peak VO2, while low PCS scores and low TAPSE at entry were predictors of improvement in PCS scores. In conclusion, extensive BPA led to improved exercise tolerance and physical QOL scores, even in CTEPH patients with partially improved hemodynamics.

## Introduction

Patients with chronic thromboembolic pulmonary hypertension (CTEPH) with poorly controlled pulmonary arterial pressure (PAP) have a poor prognosis because these are associated with right heart failure [1–3]. In addition, it can produce several symptoms along with reduced exercise tolerance, leading to significantly impaired quality of life (QOL) [4, 5]. Although recent developments in pharmacotherapy have shown good improvements in exercise capacity and clinical prognosis [6–8], pulmonary endarterectomy (PEA) is the gold standard treatment for CTEPH as it is potentially curative [9], and it significantly improves exercise tolerance, QOL, and prognosis [5, 10]. Regarding surgical intervention, however, there are several concerns that must be addressed, such as the patient’s age, availability of limited surgical facilities, invasive nature of the surgery, presence of comorbidities, high probability of residual pulmonary hypertension, location of blood thrombi, and difficulties in repeated surgery [11].

Therapeutic intervention using balloon pulmonary angioplasty (BPA) combined with selective pulmonary vasodilators is an alternative treatment modality for patients with inoperable CTEPH, such as the peripheral type of CTEPH. The BPA technique was first reported by Feinstein et al. in 2001 who demonstrated the use of BPA to achieve a significant reduction in the mean PAP from 42±12 to 33±10 mmHg [12]. Subsequently, the introduction of modified procedures (e.g., in a staged fashion over multiple or separate procedures), improved modalities, and several other clinical practices have enabled safe and effective treatment [13–17]. BPA is useful in treating symptomatic (World Health Organization functional class [WHO-FC] III or IV) and inoperable CTEPH patients with a mean PAP of ≥30 mmHg or a pulmonary vascular resistance (PVR) of ≥300 dyne·s·cm^-5^ even after pharmacological treatment, and has a standard therapeutic goal of a mean PAP of <30 mmHg. In recent years, the short-term hemodynamic efficacy of extensive BPA which are targeted at as many lesions as possible beyond hemodynamic normalization has also been reported [18]. However, little is known about the relationship between the hemodynamic improvement resulting from extensive BPA in patients after partial hemodynamic improvement (mean PAP < 30mmHg) and exercise tolerance or QOL.

This study aimed to investigate whether objective exercise tolerance, evaluated using cardiopulmonary exercise testing (CPX) which is regarded as the gold standard for the evaluation of exercise tolerance, and the quantitative QOL score, evaluated using the 36-item short form questionnaire (SF-36), can be improved after the extensive BPA in CTEPH patients with achieved partial hemodynamic improvement. We also aimed to elucidate the characteristics of patients who benefited from the extensive BPA.

## Materials and methods

### Patient population and ethics approval

**Fig 1:**
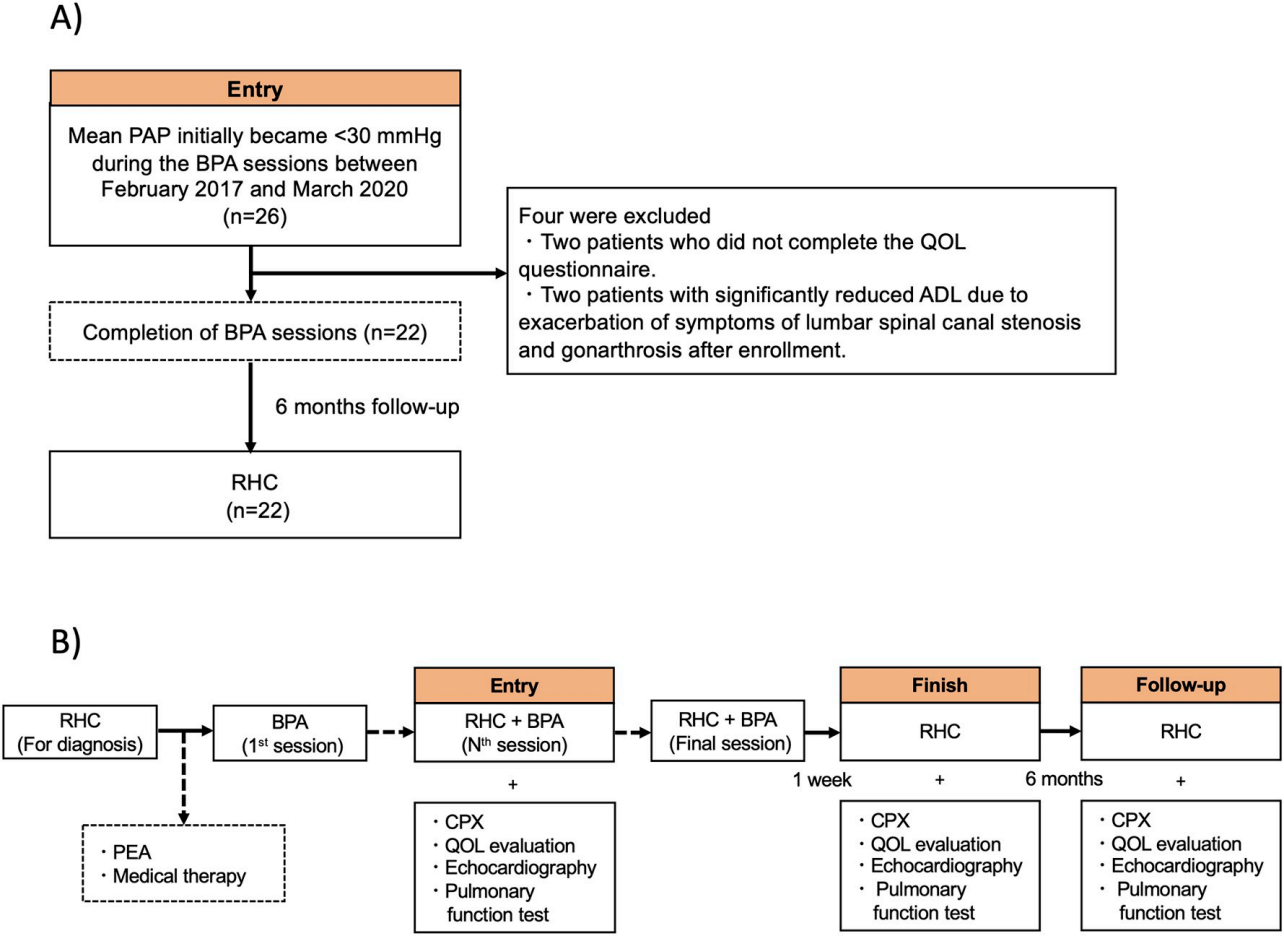
Study flowchart. (A) Patient flowchart. (B) Study design. PAP, pulmonary artery pressure; QOL, quality of life; ADL, activities of daily living; BPA, balloon pulmonary angioplasty; RHC, right heart catheterization; CPX, cardiopulmonary exercise testing; PEA, pulmonary endarterectomy.

Twenty-six consecutive patients with inoperable CTEPH who underwent BPA between February 2017 and March 2020 were enrolled when their mean PAP initially decreased to <30 mmHg during the BPA sessions (Fig 1A). Of these, 4 patients were excluded: 2 patients who did not complete the QOL questionnaire and 2 others with significantly reduced activities of daily living due to the exacerbation of symptoms of lumbar spinal canal stenosis or osteoarthritis of the knee after enrollment. Eventually, the remaining 22 patients were analyzed in this study (Fig 1A). Written informed consent from all patients were obtained, and this study was conducted as per the ethical guidelines of the Declaration of Helsinki and approved by our institutional review board (Ethics Review Subcommittee of Keio University Research Ethics Committee: permission number, 20140023).

### Study design

Right heart catheterization (RHC) was performed at diagnosis, just before each BPA session, and 1 week and 6 months after the final BPA session. Additionally, CPX, QOL evaluation using the SF-36, transthoracic echocardiography, and pulmonary function test were performed at enrollment (entry), just after the final BPA session (finish), and at the 6-month follow-up (follow-up) (Fig 1B).

### Demographic data and examinations

Clinical data regarding patients’ age, sex, body mass index, home oxygen therapy status, medical history, and medications were collected. Two-dimensional echocardiographic data including the left ventricular ejection fraction (LVEF) and tricuspid annular plane systolic excursion (TAPSE), pulmonary function tests including the vital capacity (%VC) and forced expiratory volume in 1 second expressed as a percentage (FEV1%), and blood-sampling data including hemoglobin (Hb) and brain natriuretic peptide (BNP) levels were recorded at entry, finish, and follow-up.

### Right heart catheterization and balloon pulmonary angioplasty

The detailed RHC and BPA procedures have been described in our previous reports [14, 19–21]. RHC was performed in all patients to assess the right atrial pressure (RAP), pulmonary artery pressure (PAP), pulmonary capillary wedge pressure (PCWP), cardiac index (CI), pulmonary vascular resistance (PVR), mixed venous oxygen saturation (SvO2), and arterial oxygen saturation (SaO2). All RHC procedures were performed under the same conditions in breathing room air with or without oxygen therapy. BPA was divided into several sessions to prevent the complications of pulmonary reperfusion injury. The therapeutic goal of BPA was to treat the lesions as extensively as possible, in addition to normalizing pulmonary artery pressure.

### Cardiopulmonary exercise testing protocol

The patients performed the CPXs in the upright position on an electronically braked ergometer (Strength Ergo 8; Mitsubishi Electric Engineering Company, Tokyo, Japan). The exercises were performed using a ramp (10–15 W/min) protocol ergometer, according to the exercise capacity of each patient. The subjects exercised with progressive intensity until they could no longer maintain the pedaling rate (volitional exhaustion). The expired gas flow was measured using a breath-by-breath automated system (Vmax, Nihon Kohden, Tokyo, Japan, and Aeromonitor^®^, MINATO Medical Science CO., LTD., Osaka, Japan). Respiratory gas exchange including ventilation, oxygen uptake (VO_2_), and carbon dioxide (CO_2_) production were monitored continuously and measured using a 30-second average. The anaerobic threshold (AT) was determined conventionally using the procedures described by Gaskill et al. (i.e., the ventilatory equivalent, excess CO_2_, and modified V-slope methods) [22]. The peak VO_2_ was calculated as the average oxygen consumption during the last 30 seconds of exercise. The minute ventilation to carbon dioxide production (VE-VCO_2_) slope was based on data recorded from the onset of exercise to the respiratory compensation point, and it was obtained by performing linear regression analysis of data acquired throughout the period of exercise [23, 24].

### QOL evaluation

We used the Japanese SF-36v2 (version 2) questionnaire for QOL assessment. It is composed of 8 subscales: physical functioning (PF), role-physical (RP), bodily pain (BP), general health (GH), vitality (VT), social functioning (SF), role-emotional (RE), and mental health (MH). The physical summary scores (physical component summary: PCS) and mental summary scores (mental component summary: MCS) were calculated by weighting the regression coefficients obtained from data of the general population. The subscales and summary scores were based on norms with a mean of 50 and a standard deviation of 10. This standard was established based on a study of the healthy Japanese population [25].

### Statistical analysis

We evaluated the characteristics including patients’ demographics at entry. We also evaluated data of the RHC and CPX tests and QOL questionnaires at entry, finish, and follow-up. Continuous variables are presented as mean±standard deviation (SD), and categorical variables are expressed as numbers and percentages. Unless otherwise indicated, BNP levels are reported as median and interquartile range (IQR). Multiple comparisons of the parameters of RHC and CPX, and the subscale and summary scores of the SF-36 at entry, finish, and follow-up were evaluated using Friedman’s test. Linear regression analysis was performed to determine the factors associated with a degree of improvement in the peak VO_2_ and QOL evaluation from entry to follow-up. Coefficients are shown with 95% confidence intervals (CIs) and standard error. All *P*-values were two-sided with a significance threshold of *P*<0.05. Statistical analysis was performed using IBM SPSS Statistics 24.0 (IBM Corp., Armonk, NY).

## Results

### Baseline characteristics

The baseline characteristics of the patients are summarized in Table 1. The average age was 66±10 years, and 59% of the patients were female. At entry, 47%, 8%, and 0% patients had received soluble guanylate cyclase (sGC) stimulators, prostaglandin I_2_ (PGI_2_) analogues, and phosphodiesterase type 5 (PDE-5) inhibitors, respectively. The changes in the medical treatment and demographic data are shown in Table 2 and S1 Table, respectively. Fifteen patients (68%) had received home oxygen therapy (HOT) prior to the first BPA session, 3 discontinued the HOT prior to entry, and at the follow-up, the 3 remaining patients received HOT. Oral anticoagulants were appropriately prescribed to all patients during the study.

**Table 1:** Patient characteristics.

	Entry
Age, years	66±10
Female	13 (59%)
Body mass index, kg/m^2^	22±2.1
**Medical history**	
Hypertension	8 (36%)
Dyslipidemia	4 (18%)
Diabetes mellitus	1 (4%)
**Medical treatment**	
ER antagonist	2 (9%)
PDE-5 inhibitor	0
sGC stimulator	11 (47%)
PGI_2_ analogues	2 (8%)
Oral anticoagulant	22 (100%)
Home oxygen therapy	12 (54%)
**Pulmonary function test**	
%vital capacity, %	101±16
Forced expiratory volume 1%, %	69±7
**Echocardiography data**	
LVEF, %	65±4
TAPSE	1.9± 0.3
**Laboratory data**	
Hemoglobin, g/dL	13.1±1.8
Brain natriuretic peptide (BNP), pg/ml	34 (12–61)

ER antagonist; endothelin receptor antagonist, sGC stimulator; soluble guanylate cyclase stimulators, PGI_2_ analogues; prostaglandin I_2_ analogues, LVEF; left ventricular ejection fraction, TAPSE; tricuspid annular plane systolic excursion.

**Table 2:** Changes of medical treatment data.

	Prior to BPA	Entry	Finish	Follow-up
ER antagonist	2 (9%)	2 (9%)	1 (5%)	1 (5%)
PDE-5 inhibitor	1 (4%)	0	1 (4%)	1 (5%)
sGC stimulator	7 (31%)	11 (47%)	11 (47%)	10 (45%)
PGI_2_ analogues	3 (13%)	2 (8%)	0	0
Oral anticoagulant	22 (100%)	22 (100%)	22 (100%)	22 (100%)
Home oxygen therapy	15 (68%)	12 (54%)	4 (18%)	3 (14%)

BPA; balloon pulmonary angioplasty, ER antagonist; endothelin receptor antagonist, PDE-5 inhibitor; phosphodiesterase type 5 inhibitor, sGC stimulator; soluble guanylate cyclase stimulator, PGI_2_ analogues; prostaglandin I_2_ analogues.

### BPA procedures and hemodynamic data

Table 3 shows the changes in the hemodynamic parameters prior to the first BPA session, and at entry, finish, and follow-up. The patients had already undergone 3.1±1.3 sessions of BPA at entry, and a total of 6.4±1.5 sessions were performed until completion (point of finish). Extensive BPA significantly improved the mean RAP, mean PAP, PVR, SvO_2,_ and SaO_2_. These hemodynamic improvements were maintained for 6 months after the final BPA session despite the use of nearly identical selective pulmonary vasodilators from entry to follow-up.

**Table 3:** Trend of parameters of right heart catheterization.

	Prior to BPA	Entry	Finish	Follow-up	*P* value
Mean RAP, mmHg	5.0±4.2	1.0±1.7	0.8±1.2	1.5±1.4	**0.041**
Mean PAP, mmHg	42±11	27±1	18±3	20±3	**<0.001**
PCWP, mmHg	6.7±2.8	5.8±3.5	5.3±2.5	6.5±2.4	**0.086**
CI	2.2±0.5	2.5±0.5	2.4±0.3	2.6±0.5	**0.711**
PVR, dynes*sec*cm^−5^*m^2^	861±402	455±127	279±70	248±89	**<0.001**
SvO_2,_ %	63±7	68±5	71±3	72±4	**0.022**
SaO_2_, %	89±4	91±4	95±2	94±2	**0.005**
Total BPA sessions	0	3.1±1.3	6.4±1.5	6.4±1.5	-

BPA; balloon pulmonary angioplasty, RAP; right atrial pressure, PAP; pulmonary artery pressure, PCWP; pulmonary capillary wedge pressure, CI; cardiac index, PVR; pulmonary vascular resistance, SvO_2_; mixed venous oxygen saturation, SaO_2_; arterial oxygen saturation.

### Changes in the parameters of exercise tolerance and QOL evaluations

**Fig 2:**
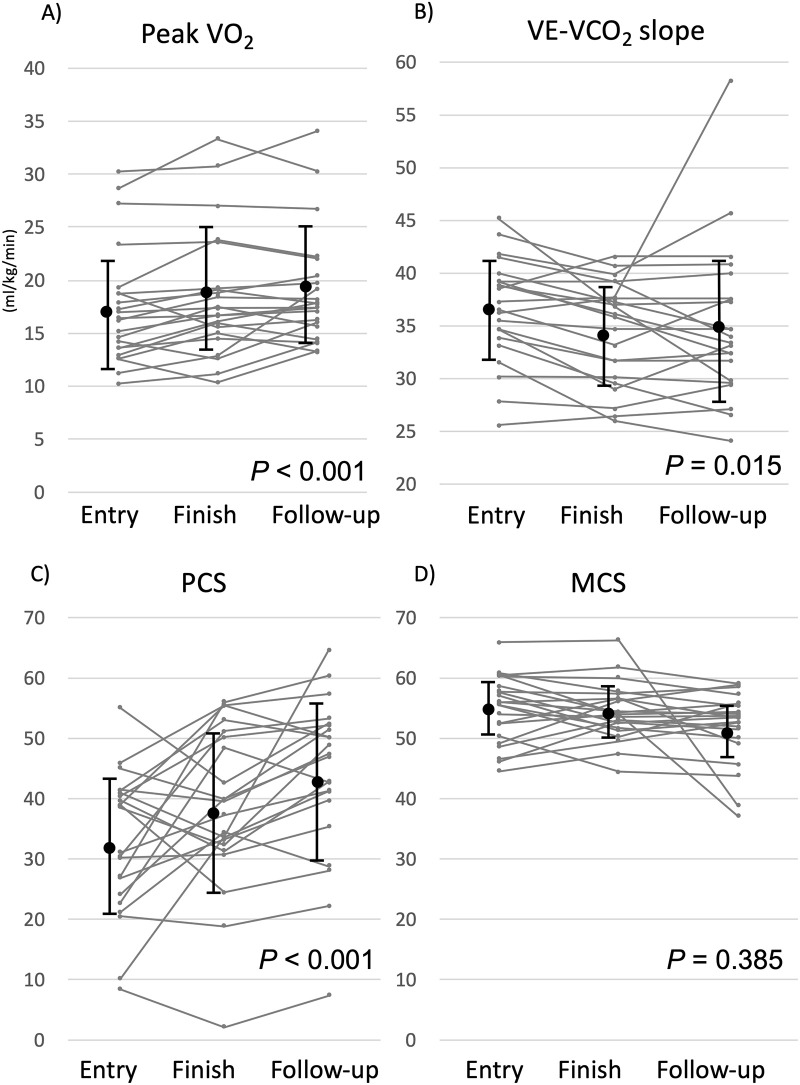
A-D. Trends depicting the peak VO_2_, VE-VCO_2_ slope, PCS, and MCS. VO_2_, oxygen intake; VE-VCO_2_ slope, ventilation/carbon dioxide slope; PCS, physical component summary; MCS, mental component summary.

Tables 4 and 5 show the changes in the parameters of exercise tolerance and QOL evaluations, respectively. The peak VO_2_ showed a significant improvement at entry, finish, and follow-up (17.3±5.5, 18.4±5.9, and 18.9±5.3 mL/kg/min, respectively; *P*<0.001; Fig 2A). In addition, extensive BPA positively affected the improvement in the VE-VCO_2_ slope (36±4, 34±5, and 35±7, respectively, *P* = 0.015; Fig 2B). Regarding the QOL, the PCS score at entry (32±11) was worse than that in the healthy Japanese population, but the MCS score was preserved (55±4). After a series of extensive BPA sessions, an improvement in the subscale scores of PF (33±13, 39±14, and 42±13, respectively, *P* = 0.002), RP (35±11, 40±13, and 44±11, respectively, *P* = 0.002), GH (42±7, 46±9, and 47±7, respectively, *P* = 0.005), and SF (35±14, 43±10, and 48±9, respectively, *P* = 0.005) was observed. In addition, PCS scores significantly improved (32±11, 38±13, and 43±13, respectively, *P*<0.001, Fig 2C), but MCS scores exhibited a non-significant change (55±4, 54±4, and 51±5, respectively, *P* = 0.385, Fig 2D).

**Table 4:** Trend of parameters of cardiopulmonary exercise test.

	Entry	Finish	Follow-up	*P* value
**Rest**				
SpO_2,_ %	95±2	96±1	96±1	**0.108**
VO_2_, ml/kg/min	4.0±0.65	3.7±0.42	3.9±0.55	**0.554**
**AT**				
SpO_2,_ %	91±4	93±3	94±2	**0.002**
VO_2_, ml/kg/min	11.6±2.6	12.0±2.5	12.8±2.9	**0.108**
**Peak**				
SpO_2,_ %	89±5	91±3	92±3	**<0.001**
VO_2_, ml/kg/min	17.3±5.5	18.4±5.9	18.9±5.3	**<0.001**
WR, Watt	68±31	77±36	79±36	**<0.001**
RQ	1.13±0.09	1.14±0.11	1.12±0.11	**0.529**
**Other parameters**				
ΔVO_2_/ΔWR	9.3±1.7	9.2±1.6	9.8±1.9	**0.496**
VE-VCO_2_ slope	36±4	34±5	35±7	**0.015**

SpO_2_; percutaneous arterial oxygen saturation, VO_2_; oxygen intake, AT; anaerobic threshold, WR; work ratio, RQ; respiratory quotient, VE-VCO_2_ slope; ventilation/carbon dioxide slope.

**Table 5:** Trend of SF-36 questionnaires.

	Entry	Finish	Follow-up	*P* value
PF (physical functioning)	33±13	39±14	42±13	**0.002**
RP (role-physical)	35±11	40±13	44±11	**0.002**
BP (bodily pain)	50±10	50±8	49±9	**0.743**
GH (general health)	42±7	46±9	47±7	**0.005**
VT (vitality)	47±11	49±9	49±11	**0.392**
SF (social functioning)	35±14	43±10	48±9	**0.005**
RE (role-emotional)	41±11	44±11	48±10	**0.072**
MH (mental health)	47±10	50±9	49±11	**0.098**
PCS (physical component summary)	32±11	38±13	43±13	**<0.001**
MCS (mental component summary)	55±4	54±4	51±5	**0.385**

### Predictors of improved exercise tolerance and PCS scores

First, we analyzed the factors that contributed to the improvement in exercise tolerance. Linear regression analysis revealed that age (coefficient [coeff]: 8.23 per 10-year increment, [95% confidence interval {CI}: 1.65, 14.8], *P* = 0.017), and a low peak VO_2_ at entry (coeff: -1.32, [95% CI: -2.60, -0.01], *P* = 0.047) were predictors of improvement in the peak VO_2_. Conversely, the period from symptom onset to the first BPA session or improvement in the mean PAP were not associated with improvement in the peak VO_2_. Next, we analyzed the factors that contributed to improvement in the PCS scores. Low PCS scores at entry (coeff: -0.55, [95% CI: -1.04, -0.07]) and low TAPSE at entry (coeff: -22.0, [95% CI: -41.8, -2.13], *P* = 0.032) were predictors of improvement in the PCS scores (Table 6).

**Table 6:** Predictors associated with improvement of peak VO_2_ and PCS.

	peak VO_2_			PCS		
	Coefficient (95% Confidence interval)	Standard error	*P* value	Coefficient (95% Confidence interval)	Standard error	*P* value
Age (per 10 y-increase)	8.23 (1.65, 14.8)	3.15	**0.017**	-2.67 (-8.69, 3.33)	2.88	**0.364**
Female	0.51 (-15.3, 16.4)	7.61	**0.947**	4.8 (-7.7, 17.5)	6.05	**0.429**
Time from symptom onset to first BPA session (months)	-0.01 (-0.18, 0.15)	0.08	**0.872**	-0.03 (-0.17, 0.09)	0.06	**0.548**
Discontinuation of HOT	9.23 (-6.41, 24.8)	7.50	**0.233**	-7.29 (-19.9, 5.36)	6.07	**0.244**
BNP at entry	0.08 (-0.11, 0.28)	0.09	**0.403**	-0.08 (-0.24, 0.08)	0.07	**0.305**
PCS at entry	-0.02 (-0.70, 0.66)	0.32	**0.951**	-0.55 (-1.04, -0.07)	0.23	**0.025**
MCS at entry	-0.63 (-1.50, 1.38)	0.69	**0.928**	0.95 (-0.12, 2.03)	0.51	**0.080**
Mean PAP at entry	-2.48 (-6.75, 1.78)	2.04	**0.238**	0.67 (-2.88, 4.23)	1.70	**0.698**
Δ mean PAP (= entry - finish)	-0.03 (-1.82, 1.75)	0.85	**0.966**	-0.30 (-1.74, 1.13)	0.68	**0.660**
CI at entry	7.03 (-6.46, 21.0)	6.60	**0.282**	-2.77 (-14.1, 8.60)	5.45	**0.617**
SaO_2_ at entry	87.1 (72.7, 101)	6.93	**0.526**	91.2 (88.4, 94.0)	1.34	**0.727**
ΔSaO_2_ (= finish - entry)	10.1 (-2.90, 23.3)	6.28	**0.295**	3.84 (1.26, 6.43)	1.24	**0.668**
PVR at entry	-0.03 (-0.09, 0.02)	0.02	**0.294**	-0.02 (-0.07, 0.02)	0.02	**0.360**
TAPSE at entry	-22.9 (-49.6, 3.68)	12.5	**0.086**	-22.0 (-41.8, -2.13)	9.32	**0.032**
Peak VO_2_ at entry	-1.32 (-2.60, -0.01)	0.62	**0.047**	0.07 (-1.09, 1.23)	0.56	**0.900**
ΔSpO_2_ at Peak (= finish - entry)	0.70 (-7.94, 9.34)	4.14	**0.626**	2.91 (1.25, 4.58)	0.80	**0.719**
VE-VCO_2_ slope at entry	0.51 (-1.10, 2.12)	0.77	**0.514**	0.14 (-1.16, 1.46)	0.63	**0.818**

VO_2_; oxygen intake, BPA; balloon pulmonary angioplasty, HOT; home oxygen therapy, BNP; brain natriuretic peptide, PCS; physical component summary, MCS; mental component summary, PAP; pulmonary artery pressure, CI; cardiac index, SaO_2_; arterial oxygen saturation, SpO_2_; percutaneous arterial oxygen saturation, PVR; pulmonary vascular resistance, TAPSE; tricuspid annular plane systolic excursion, VE-VCO_2_ slope; ventilation/carbon dioxide slop.

## Discussion

The most striking result to emerge from the data was that an extensive BPA in inoperable CTEPH patients after partial hemodynamic improvement is associated with improved objective exercise tolerance, evaluated using CPX, particularly in elderly patients or patients with reduced exercise capacity. Furthermore, an improvement in the QOL in terms of the physical function was also observed 6 months after extensive BPA, in addition to the improvement observed just after the final BPA session.

It has been reported that conventional BPA, which has a standard therapeutic goal of a mean PAP of <30 mmHg, has been shown to improve exercise tolerance [13, 26]. In addition, improved exercise intolerance was also observed not only just after BPA but also at the 3-month follow-up [15, 27]. In our study, the additional extensive BPA therapy could potentially improve the exercise intolerance even under highly restrictive conditions where the mean PAP was <30 mmHg. Further, a favorable finding was observed at the short-term follow-up (at 6 months). The hemodynamic improvement obtained after extensive BPA as regards the exercise tolerance may be noteworthy because a higher level of physical fitness may extend healthy life expectancy [28, 29]. An improvement of approximately 9% in the peak VO_2_ values (from 17.3 to 18.9 mL/kg/min) was noted, which may be a satisfactory clinical effect. Improved exercise tolerance was strongly related to increasing age and poor exercise tolerance. The impairment in the peripheral muscle adaptation, respiratory function, and physical conditioning is generally greater in older patients. Particularly, the poor cardiorespiratory function may be related to the poor exercise tolerance in older patients. Restoration of the cardiorespiratory function via BPA may have a direct and significant impact on exercise tolerance. However, in this study the degree of further improvement in the mean PAP were not associated with improvement in the exercise tolerance. While this finding may be considered a contradiction, it was similar reaction to those of previous reports, thereby suggesting that further hemodynamic improvement of CTEPH may not reflect the degree of exercise capacity [30–32]. Moreover, the duration from symptom onset to the first BPA session was a non-significant factor. This result was also consistent with the findings of a previous study [33] and suggests that BPA may improve exercise capacity regardless of the duration of onset. Although QOL questionnaires specific to patients with CTEPH have never been developed, it is desirable to evaluate QOL in multiple ways, including the physical, mental, and social aspects. The SF-36, which assesses QOL in a multidimensional manner, is widely used to assess the effectiveness of the PEA procedure [10, 34, 35]. Hemodynamically unstable CTEPH patients are known to be associated with a significant decrease in the QOL as well as a poor prognosis [4]. However, whether resolution of pulmonary artery stenosis using BPA improves the QOL remains to be fully explored. Previously, Darocha et al. reported that BPA completion could achieve a mean PAP reduction from 51.7±10.6 to 35.0±9.1 mmHg, which simultaneously resulted in a significant improvement in the PCS score from 29.5 to 39.4 and MCS score from 41 to 51.9 [36]. However, the hemodynamic improvement was inadequate compared to that reported in previous studies [13, 16–18]. Another study showed that invasive therapies (BPA in 24 patients and PEA in 15 patients) improved the PCS and MCS scores (from 23.2±16.8 to 34.0±14.6 and from 50.9±9.0 to 54.4±7.4, respectively) along with a decrease in the mean PAP from 37.6±10.7 to 22.9±7.6 mmHg; however, this study included a relatively large proportion of patients treated with PEA [33]. Unlike both these studies, our study showed that extensive BPA improved the physical QOL score in selected CTEPH patients who had mean PAP of <30 mmHg. Conversely, the mental QOL score was higher than the average mental QOL score of the Japanese population at entry; therefore, there may be no room for further improvement in the mental component despite additional hemodynamic improvement. Among the individual subscales, significant improvements were observed in the PF, RP, GH, and SF scores. The GH subscale score, in particular, showed a tendency to increase from the commencement of extensive BPA till completion, which implies an improvement in the health status through improved hemodynamics. Further improvements in the PR, RP, and SF subscale scores were observed at the 6-month follow-up. This implies that following the improved health status, the participation in social activities resulted in not only physical but also social fulfillment. Factors contributing to the improvement of the physical QOL scores after extensive BPA were low physical QOL scores at baseline and poor TAPSE values at entry. TAPSE generally reflects right ventricular function, and it has been reported that right ventricular dysfunction is associated with the physical QOL in tetralogy of Fallot and pulmonary hypertension [37, 38]. The present study also showed a mild correlation between TAPSE at entry and PCS at entry (correlation coefficient = 0.445), which suggested that right ventricular function contributed to physical QOL in CTEPH patients. Therefore, in addition to the low physical QOL at entry, poor right ventricular function at entry could also contribute to an improvement in physical QOL. The numerical amelioration in the TAPSE with extensive BPA may have improved hemodynamics during daily activity, leading to improvement in physical symptoms. To elucidate the association between right ventricular dysfunction and physical QOL in CTEPH patients undergoing extensive BPA, further investigation is warranted. For patients who perform poorly in terms of these parameters, namely, the physical QOL score or TAPSE at entry, there is room for further improvement; therefore, with a thorough evaluation of these factors, we can expect the potential for improvement in physical QOL with extensive BPA.

The present study has some limitations. First, as a single-center observational study, selection bias cannot be ruled out. Moreover, the possibility of residual confounding factors such as mental disorders, frailty, or socioeconomic status, which may explain some of the observed differences in exercise capacity or QOL, remains. Second, the follow-up period was short. The mid-term or long-term efficacy of extensive BPA in improving exercise tolerance or QOL is unknown. Third, the number of patients was small; therefore, we could not evaluate independent factors contributing to the improvement due to the lack of multivariable analysis. Our study did not include control samples for comparison. Further randomized studies with large sample sizes and a long-term follow-up are required to overcome these limitations.

In conclusion, extensive BPA was associated with improved exercise tolerance and physical QOL scores, even in CTEPH patients with relatively improved hemodynamics. This was particularly true for patients with poor physical mobility and elderly patients.

## Supporting information

S1 Table Changes of demographic data. BPA; balloon pulmonary angioplasty, TAPSE; tricuspid annular plane systolic excursion.
(DOCX)

S1 Data (XLSX)
