# Supplementary material for: Exercise tolerance and quality of life in hemodynamically partially improved patients with chronic thromboembolic pulmonary hypertension treated with balloon pulmonary angioplasty
Source: PLoS One. 2021 Jul 23;16(7):e0255180. doi: 10.1371/journal.pone.0255180 (PMC8301648; doi:10.1371/journal.pone.0255180)
Supplement: S1 Table — BPA; balloon pulmonary angioplasty, TAPSE; tricuspid annular plane systolic excursion. (DOCX) [file pone.0255180.s001.docx]

|  | **Prior to BPA** | **Entry** | **Finish** | **Follow-up** | ***P* value** |
| --- | --- | --- | --- | --- | --- |
| **Pulmonary function test** |  |  |  |  |  |
| %vital capacity, % | 97±16 | 101±16 | 104±15 | 106±16 | 0.368 |
| Forced expiratory volume 1%, % | 68±7 | 69±7 | 69±7 | 69±8 | 0.538 |
| **Echocardiography data** |  |  |  |  |  |
| LVEF, % | 68±5 | 65±4 | 65±4 | 66±4 | 0.486 |
| TAPSE, cm | 1.7± 0.3 | 1.9± 0.3 | 2.1± 0.4 | 2.1± 0.3 | 0.143 |
| **Laboratory data** |  |  |  |  |  |
| Hemoglobin, g/dL | 13.9±1.8 | 13.1±1.8 | 13.1±1.9 | 13.0±1.6 | 0.566 |
| Brain natriuretic peptide, pg/ml | 89 (46-372) | 34 (12-61) | 29 (15-41) | 30 (16-45) | 0.264 |

BPA; balloon pulmonary angioplasty, LVEF; left ventricular ejection fraction, TAPSE; tricuspid annular plane systolic excursion.
